# Universal Reversible Hydrogen Potential for Electrocatalytic Ammonia Splitting Reactions in Nonaqueous Solvents from Unified pH Measurements

**DOI:** 10.1021/acs.inorgchem.5c02177

**Published:** 2025-08-07

**Authors:** Chenjia Mi, Jaan Saame, Agnes Heering, Xiaoyin Zhang, Oluwafemi Abubakar, Ivo Leito, Thomas W. Hamann

**Affiliations:** † Department of Chemistry, 3078 Michigan State University, East Lansing, Michigan 48824-1322, United States; ‡ Institute of Chemistry, 37546 University of Tartu, Ravila 14a Str, Tartu 50411, Estonia

## Abstract

In this work, we introduce a new approach of using differential potentiometric measurements in four nonaqueous solvents—MeCN, THF, DMF, and PC—to determine the universal pH_abs_^H_2_O^ values aligned to the aqueous pH scale for dilute NH_4_^+^/NH_3_ solutions. Knowledge of the pH_abs_^H_2_O^ values allows simple determination of the reversible hydrogen potential in any given solvent relative to the aqueous standard hydrogen electrode (SHE) and, most importantly, ensures comparability across different solvents. As an independent method, Open Circuit Potenial measurements were carried out in the same solvents titrated with NH_4_^+^/NH_3_ to obtain alternative values for the reversible hydrogen potential in these solvents. The close agreement of these two methods, as well as calculated potentials from literature values when available, substantiates the new, simpler, and more robust approach to determine the reversible hydrogen potential introduced here. We further use the reversible hydrogen potential values established here to report, for the first time, the overpotential for ammonia oxidation as a function of solvent, with a recently discovered ruthenium catalyst.

## Graphical abstract

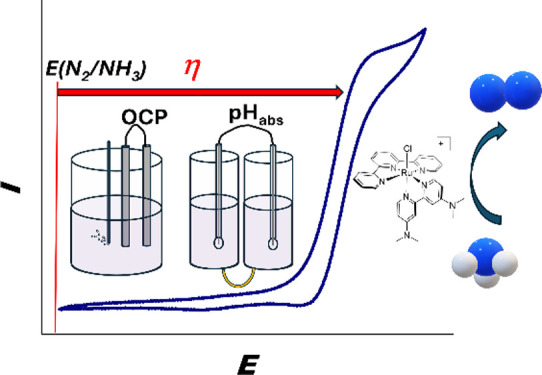

## Introduction

The overpotential, η, of electrocatalytic reactions is a key parameter in determining the chemical energy conversion efficiency. This is defined as the potential needed to sustain a reaction at a given rate (current) relative to the electrochemical potential of the reaction. Clearly, the choice of current to compare the potentials of different catalysts and solvent/reaction conditions is critical. We refer to other perspective papers that describe this half of the overpotential in detail.^1,2^ Determination of overpotential also requires knowledge of the thermodynamic electrochemical potential of the reaction of interest under the conditions of the measurement. Most fuel formation and consumption reactions of interest involve proton-coupled electron transfer (PCET) reactions. For example, there has been recent interest in utilizing ammonia as a hydrogen carrier or carbon-free renewable fuel.^4–6^ Thus, determining the overpotential of the nitrogen reduction reaction (NRR) or ammonia oxidation reaction (AOR) requires knowledge of the N_2_/NH_3_ reversible potential, *E*_N_2_/NH_3__.^7^ The lack of a catalyst capable of reversible interconversion of N_2_/NH_3_ makes the direct measurement of this potential challenging (if not impossible), and there is no example of such a measurement to the best of our knowledge. Knowledge of the hydrogen evolution electrochemical, *E*_H^+^/H_2__ potential under given reaction conditions, combined with thermochemical data, allows for the electrochemical potential for ammonia oxidation in a given reaction medium to be determined, as discussed below.^8,9^

The electrochemical potential of the hydrogen evolution reaction

$$H^{+}+e^{-}⇌\frac{1}{2}H_{2}(g)$$ 1

is given by

$$E_{H^{+}/H_{2}}={E^{{}^{\circ}}}_{H^{+}/H_{2}}-\frac{2.303RT}{2F}\log\;\frac{p(H_{2})}{p^{{}^{\circ}}}+\frac{2.303RT}{F}\log(\alpha_{H^{+}})$$ 2

where *E*°_H^+^/H_2__ is the standard potential of H^+^/H_2_ defined at 298 K, *p*(H_2_) is the partial pressure of H_2_, *p*° is the standard pressure (1 bar), and α_H^+^_is the proton activity.^10^ In aqueous solutions, under standard state conditions (α_H^+^_ = 1, *p*(H_2_) = 1 bar), *E*_H^+^/H_2__ = *E*°_H^+^/H_2__, which is defined as the standard hydrogen electrode, SHE. In aqueous conditions, *E*°_H^+^/H_2__ is defined as 0 V (SHE); however, in nonaqueous solvents, it can differ by several hundred millivolts. Under nonstandard state conditions, the hydrogen potential is referred to as the reversible hydrogen electrode, RHE. As pH is defined as

$$\mathrm{pH}=-\log(\alpha_{H^{+}})$$ 3

The RHE potential can be written as

$$E_{H^{+}/H_{2}}={E^{{}^{\circ}}}_{H^{+}/H_{2}}-\frac{2.303RT}{2F}\log\;\frac{p\left(H_{2}\right)}{p^{{}^{\circ}}}-\frac{2.303RT}{F}\mathrm{pH}$$ 4

This leads to the familiar 0.0592 V negative shift in potential with the increase of pH by one unit, as well as a 0.0296 V positive shift with each order of magnitude decrease in *p*(H_2_) from standard conditions. We note that the effect of hydrogen partial pressure on the potential is not commonly accounted for in reporting potentials vs RHE, but this can have a non-negligible effect.

In nonaqueous electrolytes, the electrochemical potential of an acid, BH^+^, to hydrogen and its conjugate base, B,

$${\mathrm{BH}}^{+}+e^{-}⇌B+\frac{1}{2}H_{2}(g)$$ 5

is generally reported using nonstandard state conditions, and is given by

$$E_{{\mathrm{BH}}^{+}/H_{2}}=E_{{\mathrm{BH}}^{+}/H_{2}}^{◦}-\frac{2.303RT}{2F}\log\;\frac{p\left(H_{2}\right)}{p^{{}^{\circ}}}-\frac{2.303RT}{F}\log\left(\frac{\left[B\right]}{\left[{\mathrm{BH}}^{+}\right]}\right)$$ 6

where *E*_^°^BH_^+^_/H2_ is the standard potential of the acid BH^+^ described by eq 5, and [B] and [BH^+^] are the concentrations of base B and its conjugate acid BH^+^, respectively (assuming unity activity coefficients). The standard potential of the acid BH^+^ can be related to the standard hydrogen potential in a given solvent, adjusted by the p*K*_aH_ of the base B (equivalent to the p*K*_a_ of BH^+^) in that solvent, as described by the thermochemical cycle in ref 11, and given by

$$E_{{\mathrm{BH}}^{+}/H_{2}}^{◦}=E_{H^{+}/H_{2}}^{◦}-\frac{2.303RT}{F}pK_{\mathrm{aH}}$$ 7

Knowledge of the standard hydrogen potential, *E*°_H^+^/H2_, and the p*K*_aH_ of a base in a given solvent allows for a straightforward estimate of the acid potential and, therefore, the nitrogen evolution potential. The application of eqs 6 and 7 was demonstrated in a seminal report by Roberts in acetonitrile (MeCN), using open-circuit potential (OCP) measurements of a series of bases with known p*K*_aH_ values.^11^ Nernstian potential shifts (59 mV/decade) with p*K*_aH_, and relative acid:base concentrations, were observed. Extrapolation of this data to a hypothetical p*K*_aH_ of 0 allowed for the determination of the standard potential, *E*_H^+^/H_2_,MeCN_^°^ = −0.028 V vs Fc^+/0^.^11^ This reported value, combined with the vast amount of thermochemical data reported in MeCN, allowed Miller and co-workers to determine the standard reduction potential of N_2_ to various proton-containing products in aqueous and MeCN solvents.^8^

OCP measurements in a small number of other organic solvents have been carried out,^12–14^ in combination with a growing library of p*K*_aH_ values in organic solvents,^15–17^ which allows estimates of hydrogen reduction potentials as a function of solvent and conjugate base referenced to Fc^+/0^. It is critical to note that both the standard hydrogen reduction potential and p*K*_a_ depend on the solvent, which inhibits direct comparison between solvents. We also note that Fc^+/0^ is the nonaqueous reference redox system of choice following the recommendation by IUPAC.^18^ It is often assumed that *E*(Fc^+/0^) is independent of solvent and electrolyte conditions; however, this is not generally the case.^19^ Thus, caution should be used in making quantitative comparisons of potentials between solvents vs Fc^+/0^. Further, while the same physics describes aqueous and nonaqueous systems, they are usually treated independently with different reference points, which makes comparisons of the thermodynamics and overpotential of electrocatalysts, as one example, quite challenging.

Recently, a universal redox scale *E*_abs_^H2O^ was introduced, where abs denotes absolute, which allows for a common basis to compare the proton chemical potential in any solvent, including water.^20^ The scale was created based on reliable determination of single-ion transfer Gibbs energies between six solvents (water, acetonitrile, propylene carbonate, dimethylformamide, ethanol, methanol) using potentiometric measurements in specially designed cells where the half-cells were filled with solutions made in different solvents. The liquid junction potential emerging between the solvents was eliminated by utilizing the ionic liquid triethylamylammonium bis­((trifluoromethyl)­sulfonyl)­imide ([N_2225_]­[NTf_2_]) as a salt bridge electrolyte. This ionic liquid has been demonstrated to be close to ideal for that purpose,^21^ owing to the nearly identical diffusion of anion and cation. Having accurate transfer Gibbs energies of ions enabled expressing the standard potentials of a number of cells on a common scale anchored to the aqueous SHE, i.e., SHE = 0 V vs *E*_abs_^H2O^. The standard hydrogen potential can still vary by hundreds of millivolts between solvents, but use of *E*_abs_^H2O^ as a reference allows direct comparisons across solvents.

Putting the hydrogen potentials on an absolute scale allows for the introduction of the absolute reversible hydrogen potential, *E*_BH^+^/H_2__ vs *E*_abs_^H2O^, which can be used to provide a common reference that is intuitively the same as the well-known aqueous RHE, that is relevant to electrochemical proton-coupled electron-transfer reactions:

$$E_{{\mathrm{BH}}^{+}/H_{2}}=E_{H^{+}/H_{2}}^{◦}-\frac{2.303RT}{F}{\mathrm{pH}}_{\mathrm{abs}}^{H2O}$$ 8

Because the absolute scale is anchored to the SHE, *E*_H^+^/H_2__^°^ = 0 V vs *E*_abs_^H2O^. This expression for the reversible hydrogen potential of an acid in any solvent is simplified by the use of the absolute scale; however, it introduces the need to measure pH in a way that is comparable between solvents. Thanks to recent efforts, that possibility exists, via the so-called “unified pH”, pH_abs_.^22,23^ pH_abs_ is defined via the absolute chemical potential of the solvated proton and uses a universal solvent-independent standard state, the proton gas at 1 bar^24^ with pH_abs_ = 0 by definition. The most convenient way of utilizing the pH_abs_ concept is to express the unified pH values as “aligned” to the conventional aqueous pH scale, that is, expressed as pH_abs_^H2O^ values.^25^ With this approach, any medium/solvent with a pH_abs_^H2O^ value of 7.00 has the same chemical potential of the solvated proton as an aqueous solution with a conventional pH of 7.00. Thus, pH_abs_^H2O^ values have a strict thermodynamic foundation and are at the same time directly comparable between any solvents/media.^22^ The measurement method of pH_abs_^H2O^ based on differential potentiometry and anchoring to the conventional aqueous pH scale has been developed recently.^23,26^

In this work, we carry out a series of pH_abs_^H2O^ measurements of dilute equimolar solutions of NH_4_^+^/NH_3_, which allows direct determination of *E*_NH_4_^+^/H_2__^°^ vs *E*_abs_^H2O^ by eq 8 for four nonaqueous solvents: MeCN, tetrahydrofuran (THF), propylene carbonate (PC) and dimethylformamide (DMF). We then carry out a series of OCP measurements by titrating NH_3_ into electrolytes composed of the conjugate acid, NH_4_OTf, where the OTF is trifluoromethanesulfonate, in the same solvents, which allows calculations of *E*_NH_4_^+^/H_2__^°^ vs Fc^+/0^. These extrapolated values are compared with calculations based on eq 7 using literature values of the standard hydrogen potential and p*K*_aH_ values in each solvent, if known. The results of the two methods are then compared from knowledge of *E*(Fc^+/0^) vs *E*_abs_^H2O^, which allows correcting the measured standard potentials by the OCP method to the absolute scale. We find good agreement between the two methods overall, which we discuss in addition to discrepancies and potential sources of error. Thermochemical corrections allow the standard potentials of AOR, *E*_N_2_/NH_3__^°^, to be calculated from the *E*_NH_4_^+^/H_2__^°^ potentials for each solvent. In this work, H_2_ and N_2_ are always considered in the gas phase, as described in the relevant half reactions shown, and the remaining species are all solvated. Results of electrocatalytic AOR measurements are also presented, and overpotentials as a function of solvent using both the OCP method and the newly introduced absolute method are reported and discussed.

## Methods

The measurements of pH_abs_^H_2_O^ values^22^—unified pH values (pH_abs_ values) aligned to the aqueous pH scale^25^—were carried out at 25 °C, using the differential potentiometric measurement procedure described earlier:^23^ ΔpH_abs_^H_2_O^ values between two solutions were obtained from measuring the potential difference between two glass electrodes immersed in these two solutions, connected via a salt bridge filled with triethylamylammonium bis­((trifluoromethyl)­sulfonyl)­imide [N_2225_]­[NTf_2_] ionic liquid.^21^ Water content in solvents was the following: MeCN, DMF, PC: < 5 ppm; THF < 1 ppm. In order to avoid contamination of the measured solutions with water from the ambient air, all measurements were carried out in a glovebox filled with argon that was constantly circulated through purifiers for the removal of water vapor and oxygen. Metrohm 713 pH meter and Keysight B2987A electrometer with dual indicator electrode input were used. Solid-contact glass electrodes EST-0601 (Izmeritelnaya Tekhnika, serial no. 137958 and 137954) were used as hydrogen ion-sensitive electrodes. Pt wire was used as an auxiliary electrode. A self-made Faraday shield was used to reduce electrostatic noise.

OCP measurements generally followed literature methods,^11^ and a detailed description is provided in the Supporting Information. Briefly, a Pt disk working electrode, a homemade Ag^+^/Ag double junction reference electrode, and a glassy carbon counter electrode were immersed in a homemade airtight electrochemical cell. Dry H_2_ gas was passed through a bubbler-sized gas washer containing a corresponding dry solvent and introduced to the cell oriented toward the working electrode. The temperature was monitored with a thermocouple. After the OCP between WE and RE reached a stable value, the titrant was transferred to the cell with a graduated syringe with a stop valve, and the OCP was measured over ∼30 s following each addition. The reported OCP was determined by averaging the collected data within each sampling time, and the uncertainty estimate was given by the standard deviation. Before, during, and after OCP titration experiments, cyclic voltammograms (CVs) of internal references were measured to calibrate the reference electrode potential and ensure stability.

The catalyst [Ru­(tpy)­(dmabpy)­Cl]Cl was prepared using a previously reported procedure.^27^ Cyclic voltammograms as a function of scan rate were measured for a 2.5 mM solution of [Ru­(tpy)­(dmabpy)­Cl]Cl in DMF, MeCN, PC, and THF with 0.1 M NH_4_OTf as a supporting electrolyte. NH_3_ was introduced by purging until a saturation was established. All electrochemical experiments were carried out at room temperature using an autolab potentiostat and a glassy carbon disc working electrode, a custom silver/silver chloride reference electrode separated from the solution using a Pt wire embedded in a glass tube following a previously reported method,^28^ and a platinum mesh counter electrode. The reference electrode was corrected to the standard ferrocene/ferrocenium couple in each solvent by the measured parameters given in Table S5.

## Results

### Measurements of pH_abs_^H_2_O^ Values

The measurements of pH_abs_^H_2_O^ values were carried out at 298 K, using the differential potentiometric measurement procedure described earlier:^23,26^ ΔpH_abs_^H_2_O^ values between two solutions were obtained from measuring the potential difference between two glass electrodes immersed in these two solutions, connected via a salt bridge filled with [N_2225_]­[NTf_2_] ionic liquid. This salt has been demonstrated to essentially eliminate the effect of liquid junction potential.^21^ From a large number of ΔpH_abs_^H_2_O^ measurements, a so-called self-consistent “ladder”^23^ was composed, as shown in Figure 1. Included in the comparison are the standard aqueous buffer solutions with pH 4.0, 7.0, and 10.0, which serve as anchor points for anchoring the “ladder”. Mathematically, the ladder is an overdetermined system, and the absolute pH_abs_^H_2_O^ values for the investigated systems were found from a previously described least-squares minimization procedure.^23^ Its essence is allowing one to change the absolute pH_abs_^H_2_O^ values of the investigated solutions in such a manner that the sum of squares of discrepancies between the directly obtained ΔpH_abs_^H_2_O^ values on the one hand and the differences of the respective two assigned absolute pH_abs_^H_2_O^ values on the other hand will be minimal, while keeping the pH_abs_^H_2_O^ values of the standard buffer solutions constant.

**Figure 1:**
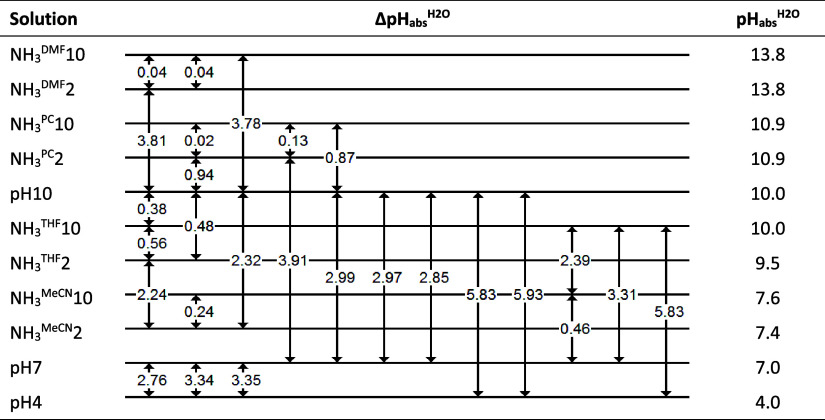
Directly measured ΔpH_abs_^H_2_O^ values are presented as “ladder” together with the assigned pH_abs_^H_2_O^ values. Numbers 10 and 2 in Sample abbreviations denote the overall buffer concentration: 10 mM (0.005 M NH_3_ + 0.005 M NH_3_HOTf) and 2 mM (0.001 M NH_3_ + 0.001 M NH_3_HOTf).

Relative pH_abs_^H_2_O^ values (ΔpH_abs_^H_2_O^ values) were measured in MeCN, THF, DMF, DCE, and PC for buffer systems composed of NH_3_ and NH_4_OTf. Two concentrations were used: 10 mM (0.005 M NH_3_ + 0.005 M NH_3_HOTf) and 2 mM (0.001 M NH_3_ + 0.001 M NH_3_HOTf). The “ladder” resulting from the ΔpH_abs_^H_2_O^ measurements, together with the absolute pH_abs_^H_2_O^ values assigned via the least-squares procedure, is presented in Figure 1. The consistency standard deviation^23^ *s* of the ladder is 0.20 pH units. This standard deviation can be interpreted as the average standard uncertainty of the obtained pH_abs_^H_2_O^ values relative to the ladder, accounting for all of the random effects and within-day systematic effects (but excluding long-term systematic effects). This uncertainty is quite high, and the assigned pH_abs_^H_2_O^ values of several systems are quite close. This means that the actual order of pH_abs_^H_2_O^ values of the systems is not necessarily exactly the same as that presented in Figure 1.

Results of pH_abs_^H_2_O^ measurements allow for the direct determination of the standard potentials vs *E*_abs_^H2O^ through eq 8. As the absolute scale is anchored to the aqueous SHE, where *E*_H^+^_^°^= 0 V by definition, the reversible NH_4_^+^/H_2_ potential in any solvent is equal to −0.0592 V × pH_abs_^H2O^. This represents a direct comparison across solvents (including water) of the proton potential of NH_4_^+^/NH_3_ electrolytes with a common reference. The averaged values of pH_abs_^H_2_O^ measurements of equimolar NH_3_:NH_4_OTf solutions are provided in Table 1 along with the corresponding reversible potentials, $E_{N{H_{4}}^{+}/H_{2}}$ , calculated using eq 8.

**Table 1:** Measured Values of pH_abs_^H2O^ of Equimolar Dilute Solutions of NH_4_^+^/NH_3_ in the Four Solvents of Interest Here^a^

solvent	pH_abs_^H_2_O^ ^b^	$E_{{{\mathrm{NH}}_{4}}^{+}/H_{2}}\;(V\;\mathrm{vs}\;E_{\mathrm{abs}}H_{2}O)$	*E*(Fc^+/0^)^c^ (V vs *E*_abs_^H_2_O^)	$E_{N{H_{4}}^{+}/H_{2}}$ ,_*corr*_ (V vs Fc+/0)^d^
MeCN	7.5	–0.444	0.488	–0.932
THF	9.75	–0.577		
PC	10.95	–0.648	0.472	–1.120
DMF	13.8	–0.817	0.432	–1.249

a Also shown are the calculated reversible hydrogen reduction potentials using eq 8, literature values of *E*(Fc^+/0^) vs *E*_abs_^H_2_O^ and the corrected RHE potentials to the Fc^+/0^ reference using eq 9.

b Average of 2 and 10 mM solutions.

c Reported values of *E*(Fc^+/0^) vs *E*_abs_^°H_2_O^ in ref 20.

d Corrected potential by reported Fc^+/0^ vs *E*_abs_^° ′ H_2_O^.

The results of $E_{N{H_{4}}^{+}/H_{2}}$ vs *E*_abs_^H_2_O^ in Table 1 cannot be directly compared with $E_{N{H_{4}}^{+}/H_{2}}$ vs Fc^+/0^ obtained from OCP measurements described below. In order to compare these values, a common reference point is needed. Fortunately, the potential of ferrocene on an absolute scale, *E*(Fc^+/0^) vs *E*_abs_^H_2_O^, has been recently reported in the solvents of interest here, with the exception of THF, and provided in Table 1.^20,29^ Thus, the measured absolute potential can simply be corrected to the ferrocene scale through the use of eq 9:

$$E_{{{\mathrm{NH}}_{4}}^{+}/H_{2}}\;\mathrm{vs}\;{\mathrm{Fc}}^{+/0}=E_{{{\mathrm{NH}}_{4}}^{+}/H_{2}}\;\mathrm{vs}\;E_{\mathrm{abs}}^{H_{2}O}-E({\mathrm{Fc}}^{+/0})\;\mathrm{vs}\;E_{\mathrm{abs}}^{H_{2}O}$$ 9

The potentials corrected to the ferrocene reference, $E_{{{\mathrm{NH}}_{4}}^{+}/H_{2},\mathrm{corr}}$ , are also given in Table 1. These values can then be compared directly with potentials calculated using eq 7, *E*_NH_4_^+^/H_2_,calc_^°^, or determined with OCP measurements, *E*_NH_4_^+^/H_2_, OCP_^°^, which are described below and given in Table 2.

**Table 2:** Values of the Literature, Values of the Standard Hydrogen Potential, and p*K*_aH_ of NH_4_^+^ Used to Calculate the Standard Potential of NH_4_^+^/NH_3_ Using eq 7 in the Four Solvents Investigated^a^

solvent	*E*_H^+^/H_2_,solv_^°^ (*V* vs Fc^+/0^)	p*K*_aH_ (NH_3_)	*E*_NH_4_^+^/H_2_, calc_^°^ (*V* vs Fc^+/0^)	*T* (K)	titration slope (V)	*E*_NH_4_^+^/H_2_, OCP_^°^ (*V* vs Fc^+/0^)
MeCN	–0.028^b^	16.46^e^	–1.002	296.0 ± 0.2	0.070	–1.019
THF	–0.343^c^	12^f^	–1.053	295.8 ± 0.1	0.066	–1.154
PC		15.9^g^		295.6 ± 0.1	0.083	–1.008
DMF	–0.620^d^	9.45^h^	–1.221	294.8 ± 0.2	0.063	–1.143

a Also, the compiled measured values are given with the Nernstian slope.

b *Inorg. Chem*., 2013, 52, 3823.

c *Chem. Rev.*, 2022, 122(1), 1–49.

d *Inorg. Chem.*, 2015, 54(24), 11883–11888.

e *J. Am. Chem. Soc.*, 1965, 87, 5005–5010.

f Calculated from p*K*_a_(NH_4_^+^) in MeCN, 16.46,^34^ and linear regression between p*K*_a_ values of protonated bases in MeCN^15^ and THF.^17^

g Izutsu, *Bull. Chem. Soc. Jpn*., 1979**.**

h *Anal. Chem.*, 1970, 42(13), 1622–1628.

### OCP Measurements

In order to compare the results of the absolute potential measurements described above, the method of Roberts was employed to perform OCP titration measurements with the NH_4_OTf/NH_3_ acid/base pair in MeCN. While this buffer is common for electrocatalytic ammonia oxidation measurements,^9,30,31^ to the best of our knowledge, the standard potential has not been measured directly. The electrolyte solution consisted of 0.3 M NH_4_OTf in MeCN, with aliquots of NH_3_ titrated in stepwise. The NH_3_ titrant was composed of a liquid formed from a 2:1 mixture of NH_3_:NH_4_OTf, termed a eurefstic.^32^ Thus, the concentration of NH_4_OTf also increases upon addition of titrant, but the change is small and was accounted for in the analysis. We found the use of a eurefstic a convenient method to control the addition of NH_3_. Decamethyl ferrocene, Me_10_Fc^+/0^, was used in place of Fc^+/0^ as an internal standard due to concerns of Fc^+^ reacting with NH_3_ and thus inhibiting stable and accurate measurements.^33^ The potentials are then corrected to *E*(Fc^+/0^) by the subtraction of 506 mV.^19^ As shown in Figure 2, a staircase pattern is observed with the injection of each aliquot of the NH_3_:NH_4_OTf titrant, indicating a stabilized OCP after each titration. Since the experiment is designed so that [NH_3_] increases while [NH_4_OTf] is held essentially constant for each addition of titrant, it is expected to see an increasing negative potential, consistent with the data plotted in Figure 2. As shown in Figure 2b, when each OCP datum is plotted against log­([NH_3_]/[NH_4_OTf]), a linear relationship is obtained with a slope of −0.070 V, close to the Nernstian slope of −0.059 V at *T* = 296.0 ± 0.2 K (measured during the OCP titration). This nearly ideal behavior allows extrapolation to the standard potential using eq 10, where [NH_3_]/[NH_4_OTf] = 1.

$$E_{\mathrm{OCP}}=E_{{\mathrm{NH}}_{4}^{+}/H_{2}}=E_{{\mathrm{NH}}_{4}^{+}/H_{2}}^{{}^{\circ}\prime }-\frac{2.303RT}{2F}\log\;p_{H_{2}}^{1/2}-\frac{2.303RT}{F}\log\;\frac{\left[{\mathrm{NH}}_{3}\right]}{\left[{\mathrm{NH}}_{4}^{+}\right]}$$ 10

**Figure 2:**
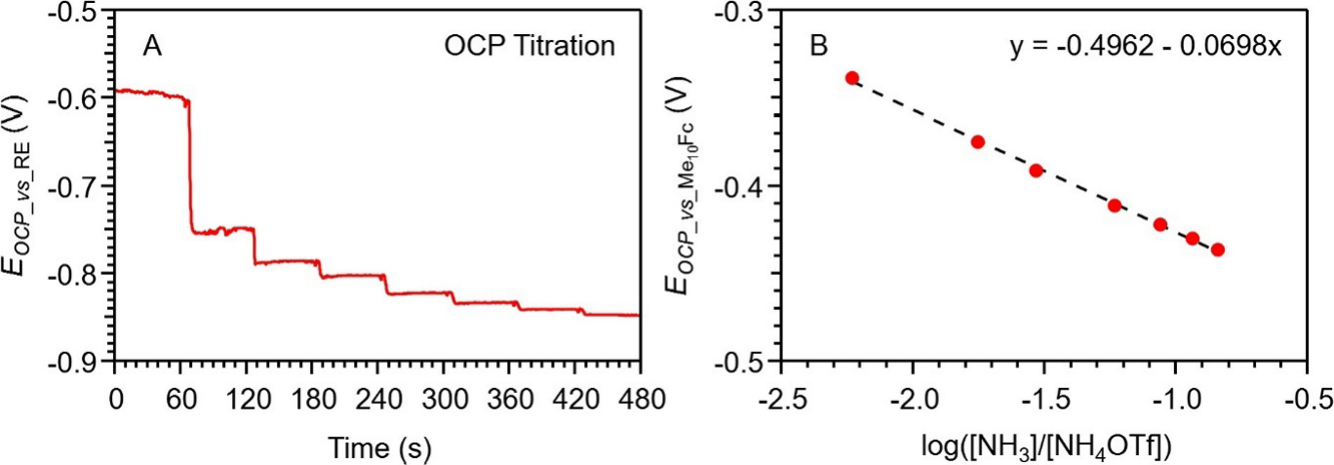
(A) OCP titration of the NH_3_–NH_4_OTf base–acid pair in MeCN. Each “stair” is recorded as an OCP datum with a corresponding concentration of NH_3_. (B) Plot of measured OCP vs log­([B]/[BH^+^]) which is fit to a straight line in accord with eq 10.

Corrections to the measured potential for any variations of the hydrogen partial pressure, *p*_H_2__^1/2^, were performed as described in Supporting Information, and used in eq 10 to yield a value of *E*_NH_4_^+^/H_2__^°^ = −1.019 V vs Fc^+/0^.

Our measured value is in good agreement with the standard potential calculated using eq 7, with the established hydrogen standard potential in MeCN, *E*_H^+^/H_2_, MeCN_^°^ = −0.028 V vs Fc^+/0^, and p*K*_aH_ of NH_4_^+^ in MeCN of 16.46,^34^ which yields a value of *E*_NH_4_^+^/H_2__^°^ = −1.002 V vs Fc^+/0^. The standard potential for ammonia oxidation to dinitrogen in MeCN can then be calculated by subtracting 60 mV to yield *E*_N_2_/NH_3__^°^ = −0.96 V vs Fc^+/0^ (measured) or *E*_N_2_/NH_3__^°^ = −0.94 V vs Fc^+/0^ (calculated). Prior reports of *E*_N_2_/NH_3__^°^ values in MeCN vary widely. Some reports used eq 7 to calculate the standard potential, with the same calculated value found here in MeCN, which was used to determine the ammonia oxidation overpotential.^35,36^ Others, however, have reported a value of −0.53 V vs Fc^+/0^ in MeCN.^37,38^ This discrepancy introduces a lot of uncertainty in prior reports of overpotential of AOR electrocatalysis carried out in MeCN, but the good agreement with reported thermochemical data and our direct measurements indicates *E*_N_2_/NH_3__^°^= (−0.95 ± 0.01) V vs Fc^+/0^ is an appropriate value to use in MeCN.

While our results are in good agreement with predictions by the Nernst equation in MeCN, which has a well-established standard potential of hydrogen and robust p*K*_a_ library, we have found that acetonitrile will tightly bind to open coordination sites on Ru catalysts following the N_2_ release step in electrocatalytic reactions, making it unsuitable as a solvent for the [Ru­(tpy)­(dmabpy)­NH_3_]^2+^ and related catalysts.^39^ It is this dead-end in the catalytic cycle that motivated us to use the less-coordinating THF as a solvent for our initial electrocatalytic measurements.^27^ We note that other reports of ammonia electrocatalysis have also employed THF as a solvent, and there is one report, discussed below, of the measurement of *E*_N_2_/NH_3__^°^ in THF.^14^ We therefore also carried out a series of OCP titration measurements with NH_3_:NH_4_OTf in THF, with the same procedure as described above, to compare with the literature report and further test the predictions of eq 7. Experimental details and data are provided in the SI. The OCP vs ln­([NH_3_]/[NH_4_OTf]) plot was linear over the region measured, with a slope of −0.0658 V, very close to that found in MeCN and in agreement with the Nernst slope of −0.059 V at *T* = 295.8 ± 0.1 K (measured during the OCP titration). Extrapolation to the standard potential and correction for hydrogen partial pressure using eq 10 produces a value of *E*_NH_4_^+^/H_2__^°^ = −1.154 V vs Fc^+/0^ in THF.

The hydrogen standard potential has been extrapolated from OCP measurements of several protonated bases in THF to produce a value of *E*_H^+^/H_2_,THF_^°^ = −0.343 V vs Fc^+/0^.^3^ The available p*K*_a_ data in THF are more limited than in MeCN, and no p*K*_a_ value for NH_4_^+^ has been reported. An estimate of the p*K*_a_ of a protonated base in THF can be made from a linear correlation of published data of organic bases in MeCN^15^ and THF.^17^ The correlation is good (*R* ^2^ = 0.98, *S* = 0.7), as has been shown previously for this pair of solvents,^16^ to produce a value of 12.0 for the p*K*_a_ of NH_4_^+^ in THF. Thus, a value of *E*_NH_4_^+^/H_2__^°^ = −1.053 V vs Fc^+/0^ can be calculated by eq 7. Note that an error in the absolute p*K*_a_ of ±1 will lead to a ±59 mV error in the estimated standard potential. The extrapolated standard potential from our measurements is ca. 100 mV more negative than the calculated value, which is more than expected. A significant (if not the main) part of the discrepancy, as noted above, may be attributed to the deviation of the estimated p*K*_a_ from the actual one. NH_3_ is a smaller molecule than any of the bases used for establishing the above-mentioned correlation, and the p*K*_aH_ of NH_3_ is therefore much more influenced by the change of solvent than the p*K*_aH_ of the bases used in the correlation. The extrapolation of the titration data, whose slope deviates slightly from that of Nernstian, will also lead to an error in the standard potential. The OCP method has been employed before to measure the standard potential of ammonium in THF, where a value of *E*_NH_4_^+^/H_2__^°^ = −0.86 V vs Fc^+/0^ was reported.^14^ While errors on the order of tens of mV are not surprising, a discrepancy of 0.2–0.3 V is significant. We note that this prior report was the result of a single measurement, as opposed to the extrapolation of a titration, which may lead to significant error. We therefore tentatively take our measured value, which is close to the calculated/predicted value, as the more accurate value, as further justified by the absolute measurements reported above and discussed below. As a result, the reported overpotentials of AOR in THF are likely underreported by ca. 200–300 mV. These results show that while conceptually simple and powerful, the OCP method is not easy to carry out accurately and consistently; in both MeCN and THF, the reported potential varies by several hundred mV. Further, the limited availability of *E*_H^+^/H_2_,solv_^°^, and p*K*_a_ values in solvents outside of water and MeCN further hinders this approach to evaluating the standard potential of PCET reactions more broadly and motivates an alternative and more general method using differential potentiometric measurements of pH_abs_^H2O^ as described above.

THF has also been proven not to be an ideal solvent for our and likely others' electrocatalysis investigations. For example, in recent work, we have found that some intermediate species of interest are insoluble, or sparingly soluble, in THF, which can be attributed to the relatively low polarity and dielectric constant.^39^ In addition, a high solution resistance hinders the accurate analysis of electrocatalysis measurements. This has motivated a wider investigation of solvents for electrocatalysis and the concomitant need to establish the thermodynamics of ammonia oxidation in solvents other than MeCN and THF. DMF is a feasible alternative with a high solubility of catalyst species of interest. Recent reports of OCP measurements of the hydrogen standard potential and a quite extensive literature of p*K*_a_ data allow for values of the ammonium standard potential to be calculated. We have recently found propylene carbonate, PC, to be a nearly ideal solvent; it is conductive, nontoxic, exhibits high solubility of catalysts, and is noncoordinating and chemically innocent. We have therefore also attempted to carry out OCP titration measurements with NH_3_:NH_4_OTf in DMF and PC in the same manner as described above. The data are provided in the SI.

The combined results of these measured values, *E*_NH_4_^+^/H_2_,OCP_^°^, are compared to the calculated values using eq 7, *E*_NH_4_^+^/H_2_,calc_^°^, for the four solvents investigated here, which are shown in Table 2. The agreement between our extrapolated standard potential values and the calculated values based on literature is generally good, with differences spanning 17–101 mV. The largest discrepancy is in THF, which is discussed above. There is no reported standard hydrogen potential from the OCP measurements in PC to allow calculation of *E*_NH_4_^+^/H_2_,calc_^°^, to compare with our measured value. Taking our measured value, however, with the known p*K*_aH_ of NH_4_^+^ in PC^40^ allows a value of *E*_H^+^/H_2_,PC_^°^ = −0.067 V vs Fc^+/0^ to be calculated with eq 7.

The values in Table 2 highlight the challenge in comparing the thermodynamics of PCET reactions across solvent systems, with large variations in hydrogen standard potentials and p*K*_aH_ values, in addition to the less obvious variation in Fc^+/0^ reference potentials. The alternate, complementary approach, which we believe is simpler and more accessible, is the use of a universal reference system instead of Fc^+/0^ and accounts for differences in proton activity in any solvent directly by using pH_abs_^H_2_O^. Use of this universal reference system to report the reversible hydrogen potential is especially appropriate for PCET electrocatalytic reactions, where the proton activity plays a central role in determining the thermodynamics of the system. We note that a complementary approach to a universal RHE has been advocated by Mayer and co-workers.^3^ In this case, potentials are reported versus H_2_(g) and can be measured with the OCP method in the specific reaction conditions. Thus, the reference potential is not identical, although very close to constant, and the OCP measurement is not as straightforward as the pH_abs_^H_2_O^. Efforts are underway in our lab to directly compare results and reconcile these reference systems.

The agreement between the $E_{{{\mathrm{NH}}_{4}}^{+}/H_{2},\mathrm{corr}}$ determined from pH_abs_^H_2_O^ measurements and *E*_NH_4_^+^/H_2_,calc_^°^ using literature values of *E*_H^+^/H_2_,solv_^°^ and p*K*_aH,solv_, is quite good: Δ*E* = 70 mV in MeCN and 28 mV in DMF. Missing reported values in PC and THF prohibit making a similar comparison for these solvents. The difference between potentials measured via the OCP method, *E*_NH_4_^+^/H_2_,OCP_^°^, and the pH_abs_^H2O^ method, *E*_NH_4_^+^/H_2_,corr_^°^, is also in reasonable agreement: Δ*E* = 87 mV in MeCN, 112 mV in PC, and 106 mV in DMF. The missing potential of ferrocene on an absolute scale in THF prohibits making a similar comparison for this solvent.

We note that the conditions of the OCP and pH_abs_^H_2_O^ measurements were not identical and may account for some of the discrepancy between these methods. The OCP measurements are carried out with 1 atm H_2_(g), or corrected for variations in *p*(H_2_); however, the pH_abs_^H_2_O^ values were not. The use of symmetric cells for pH_abs_^H_2_O^ measurements results in the difference between *p*(H_2_) in the solvents, which should cancel out, like the liquid junctions, as no H_2_ gas was introduced in either solvent. The pH_abs_^H_2_O^ measurements are performed with very dilute solutions, which represent nearly ideal conditions. There is minimal variation between concentrations of solute, with pH_abs_^H_2_O^ values of 2 and 10 mM solutions differing by an average of 0.2 pH units, which corresponds to a difference in potentials of only ca. 12 mV. Nevertheless, the difference in measured values between these two dilute solutions may indicate nonideal behavior and should be investigated further. The OCP measurements are necessarily carried out with concentrated electrolytes, however, which may exacerbate any nonideal behavior such as homoconjugation and account for some of the discrepancy between these measurements. This may be the reason for better agreement between the calculated potentials using eq 7, which utilize p*K*_aH,solv_ values determined under ideal conditions, and pH_abs_^H_2_O^ measurements compared to the OCP measurements, which contain concentrated electrolytes to minimize solution resistance and mimic electrocatalysis conditions. A larger source of error is likely due to the fact that the OCP results are extrapolated to determine the standard potential, with nonideal slopes. Further efforts are underway to further refine our protocols to more closely match the conditions of both the OCP and pH_abs_^H_2_O^ measurements and resolve any fundamental differences between the two. Given the more ideal conditions employed, and the robustness of the method demonstrated in the ladder shown in Figure 1, and the good agreement with calculations based on literature data, we believe the pH_abs_^H_2_O^ approach produces a more accurate value of $E_{N{H_{4}}^{+}/H_{2}}$ . Importantly, the comparison of potentials, or practical use of the pH_abs_^H_2_O^ determined absolute potentials, requires knowledge of a common reference point of ferrocene. All of the discussion above is based on a single set of reported values without any indication of the uncertainties associated with those values. Thus, the reported *E*(Fc^+/0^) vs *E*_abs_^H_2_O^ may be a significant source of error. Hopefully, this report will motivate further refinement of this critical reference.

### Electrocatalytic Ammonia Oxidation

The measured thermodynamic potentials for HER allow the determination of the overpotential of AOR as a function of the solvent. The detailed electrocatalytic behavior and mechanistic investigation of [Ru­(tpy)­(dmabpy)­Cl]Cl have been recently reported,^41^ and it is utilized here as it gives rise to an ideal S-shaped catalytic response conforming to an EC′ mechanism, which allows robust determination of the overpotential. The cyclic voltammograms (CV) of [Ru­(tpy)­(dmabpy)­Cl]­Cl in MeCN, THF, PC, and DMF measured vs Fc^+/0^ are provided in the SI (Figure S7). The CVs show quasi-reversible behavior in all solvents with peak-to-peak separations between 66 and 92 mV. The formal potentials span a range of ∼180 mV, from 0.117 V vs Fc^+/0^ in PC, 0.100 V vs Fc^+/0^ in MeCN, 0.077 V vs Fc^+/0^ in DMF, to −0.059 V vs Fc^+/0^ in THF. The variation in redox potentials with respect to the ferrocene potential suggests interactions of the solvent with [Ru­(tpy)­(dmabpy)­Cl]^+^ and/or [Ru­(tpy)­(dmabpy)­Cl]^2+^, such as hydrogen bonding, Lewis acid/base interactions, or pi system interactions.^19,20,29^

Figure 3 shows the CVs of [Ru­(tpy)­(dmabpy)­Cl]Cl in MeCN, PC, and DMF in the presence of saturated NH_3_. The CV in THF with saturated NH_3_ is not included in Figure 3 as nonideal electrocatalytic behavior was found in this solvent, in addition to the formation of a precipitate during measurements. We have not been able to identify the precipitate, but we have seen this behavior in THF before, which induced us to identify and investigate alternative solvents for electrocatalysis.^39^ The CVs in the presence of saturated NH_3_ exhibit the characteristic s-shaped wave of pure kinetic conditions, indicating electrocatalysis for all NH_3_-containing electrolytes.^42–44^ The CVs were also plotted vs *E*_abs_^H_2_O^ using eq 9 to convert from *E*(Fc^+/0^), however, this was not possible with THF. In order to assess the overpotential, the potential that produces 12 of the plateau current, *E*_cat/2_, was used to benchmark the electrocatalysis potential.^1,2^ The *E*_cat/2_ values were obtained by fitting the catalytic current, *i*, normalized by the anodic peak of the catalyst in the absence of substrate, *i*_p_, as previously reported.^41^ The *E*_cat/2_ varies by ca. 0.1 V between DMF, MeCN, and PC (see Table S4) when referenced to either *E*_abs_^H_2_O^ or *E*(Fc^+/0^). This is similar to the different *E*_1/2_ of the catalysts in the absence of NH_3_ in these solvents, indicating the differences in *E*_cat/2_ are primarily solvent effects on the Ru^3+/2+^ redox potential, noted above, rather than kinetic effects.

**Figure 3:**
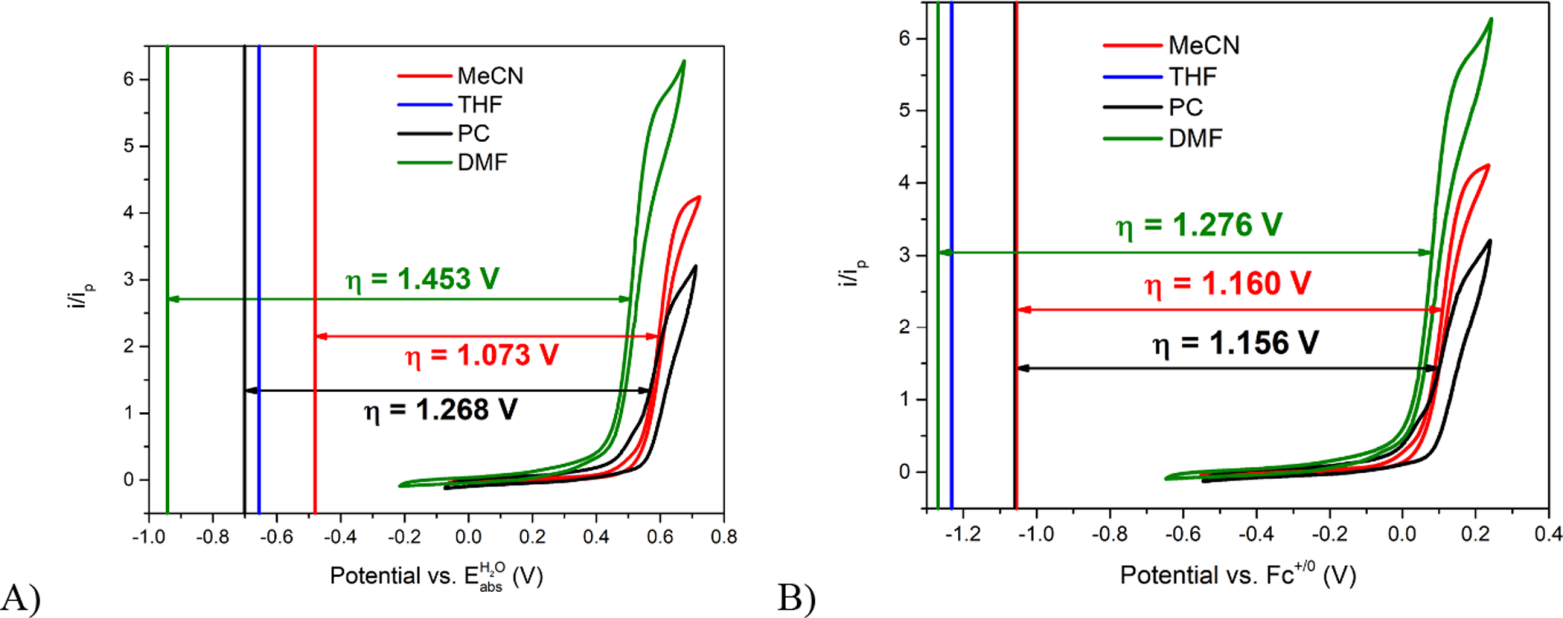
Cyclic voltammograms of 2.5 mM [Ru­(tpy)­(dmabpy)­Cl]^+^ in MeCN (red), PC (black) and DMF (green) solutions saturated with NH_3_. All electrolytes contained 0.1 M NH_4_OTf as a supporting electrolyte. (A) Reversible N_2_/NH_3_ potentials derived from pH_abs_^H_2_O^ measurements (Column 4 of Table 3) plotted as vertical lines vs *E*_abs_^H_2_O^. (B) Reversible N_2_/NH_3_ potentials derived from OCP measurements (Column 5 of Table 3) are also shown as vertical lines with potentials plotted vs Fc^+^/Fc. The overpotentials reported correspond to the double headed arrown shown. Reversible potentials in THF are also shown as a blue verticel line for reference.

The thermodynamic potentials of AOR, described by eq 11

$${\mathrm{NH}}_{3}(\mathrm{solv})-3e^{-}⇌\frac{1}{2}N_{2}(g)+3H^{+}$$ 11

were determined following a previously reported thermochemical analysis in MeCN.^8^ The solvation energy term for the transfer from gaseous NH_3_ to the different solvents is calculated from solubility measurements, described in the SI, with values provided in Table 3. The results of the standard potentials, *E*_N_2_/NH_3_,_^°^ derived from the HER potential measured with the pH_abs_^H_2_O^ (column 5 of Table 1) and OCP (column 7 of Table 2) methods, with the thermochemical cycle described elsewhere,^8^ are provided in Table 3.

**Table 3:** Values of Measured Saturated Ammonia Concentrations, Calculated Solvation Energies and Standard Potentials of N_2_/NH_3_ vs Both *E*_abs_^H_2_O^ and Fc^+/0^

solvent	[NH_3_]_sat_ (M)	solvation energy (kcal/mol)	$E_{N_{2}/{\mathrm{NH}}_{3},{pH}_{abs}^{H_{2}O}}^{{}^{\circ}}$ (*V* vs E_abs_^H_2_O^)	*E*_N_2_/NH_3_,OCP_^°^ (*V* vs Fc^+/0^)
MeCN	1.7 ± 0.1	–0.30 ± 0.03	–0.382	–0.961
THF	2.43 ± 0.01	–0.52 ± 0.01	–0.512	–1.089
PC	1.96 ± 0.01	–0.40 ± 0.02	–0.585	–0.945
DMF	3.3 ± 0.3	–0.71 ± 0.05	–0.749	–1.075

The electrocatalytic measurements were made with saturated ammonia solutions and a constant ammonium concentration in the supporting electrolyte. Given the different solubilities of ammonia, a correction to the actual reversible solution potential, *E*_N_2_/NH_3__, was made to determine the overpotential in accord with the Nernst equation:^7^

$$E_{N_{2}/{\mathrm{NH}}_{3}}=E_{N_{2}/{\mathrm{NH}}_{3}}^{{}^{\circ}}-\frac{2.303RT}{F}\log\left(\frac{\left[{\mathrm{NH}}_{3}\right]}{\left[{\mathrm{NH}}_{4}^{+}\right]}\right)$$ 12

Thus, the 17–33-fold excess of [NH_3_] to [NH_4_^+^] for the four solvents results in a 98–193 mV negative shift in potential, respectively, which are shown as vertical lines in Figure 3. The differences in thermodynamic potential between solvents have a much larger effect on the overpotential than differences in *E*_cat/2_. The effect of solvent on the reaction mechanism and rate that gives rise to *E*_cat/2_ is being actively investigated in our lab and beyond the scope of this work. There is some discrepancy, as noted above, between the magnitudes of the measured potentials using the OCP and pH_abs_^H_2_O^ methods. The overall trends, however, are very similar. Both pH_abs_^H2O^ and OCP methods indicate that DMF has the most negative thermodynamic potential for the AOR and thus between a 100–300 mV larger overpotential compared to the same catalyst in MeCN. The *E*_cat/2_ results in THF were not included in the analysis due to nonideal behavior noted above, but given the correlation between *E*_1/2_ and *E*_cat/2_ for the other solvents, we can use *E*_1/2_ to approximate the overpotential of AOR in THF. The *E*_1/2_ of [Ru­(tpy)­(dmabpy)­Cl]^+^ is approximately 150 mV more negative in THF than MeCN, while the thermodynamic potential measured by OCP is approximately 135 mV more negative in THF than MeCN. Thus, the AOR overpotential in THF is expected to be similar compared to the same catalyst in MeCN. Unfortunately, there is no way to correct the pH_abs_^H_2_O^ measurements to the *E*(Fc^+/0^) scale or the electrocatalysis results to the *E*_abs_^H_2_O^ scale in THF to enable corroboration of this finding.

## Conclusions

A series of OCP measurements were carried out in NH_4_^+^/NH_3_ containing solutions in a series of solvents to determine the thermodynamic potentials of the HER and AOR. In addition, a new method was introduced to measure the thermodynamic potentials in nonaqueous solvents through the use of pH_abs_^H2O^ measurements. The advantage of the new method is that it is a direct determination of the reversible hydrogen potential in any solvent, including aqueous solutions. It thus bridges the gap between reports in aqueous and nonaqueous electrolytes, which are often treated independently on different electrochemical scales. The agreement between these two methods, as well as calculated values based on the literature data, is generally good. Further refinement of the methodology is needed, however, in a larger variety of conditions (concentrations, solvents, and bases) to elucidate and minimize sources of error or discrepancy between the methods investigated. In addition, comparison of absolute potentials determined with the pH_abs_^H2O^ method with literature data and measurements in nonaqueous solvents, which are generally reported vs *E*(Fc^+/0^), requires knowledge of *E*(Fc^+/0^) vs *E*_abs_^H_2_O^. That data set is currently very limited but hopefully will expand rapidly to facilitate practical utilization of the absolute potential scale.

Establishment of the thermodynamic potential for AOR in different nonaqueous solvents allowed for the determination of electrocatalytic overpotential as a function of solvent. The solvent was found to have a significant effect on overpotential; while there are some differences in *E*_cat/2_, most of the variation in overpotential between solvents is due to the thermodynamic potentials. The reason for this is still not clear, but the observation that DMF gives rise to the most negative reduction potentials may come from its relatively strong Lewis base character, which can stabilize protons. Ongoing measurements in a greater variety of acids and solvents under different conditions are being carried out to elucidate this and other key factors systematically. While there are sources of error in the calculated and measured thermodynamic potentials in THF, we expect that error to be smaller than the 165 mV difference in overpotential between THF and MeCN. Other properties of THF, notably poor solubility of catalysts and intermediates, are not ideal, however. As PC has similar thermodynamic advantages as THF, with good solubility, conductivity, nontoxicity, coordination, and chemical innocense, this is the most attractive solvent we have identified to date for AOR electrocatalysis and has become the solvent of choice in our laboratory. Finally, we note this result, where the choice of nonaqueous solvent can have such a large effect on overpotentials, is not obvious. We note that only measuring or reporting electrocatalytic results vs *E*(Fc^+/0^) as an internal standard would obscure this effect, and that the accurate determination of the thermodynamic potential is critical in reporting overpotential.

## Supplementary Material



## Notes

Additional experimental details, additional results of OCP measurements, electrocatalyst characterization, and thermochemical analysis results (PDF)
