# Supplementary material for: Universal Reversible Hydrogen Potential for Electrocatalytic Ammonia Splitting Reactions in Nonaqueous Solvents from Unified pH Measurements
Source: Inorg Chem. 2025 Aug 7;64(32):16423–32. doi: 10.1021/acs.inorgchem.5c02177 (PMC12365871; doi:10.1021/acs.inorgchem.5c02177)
Supplement: Supplementary file 1 [file ic5c02177_si_001.pdf]

Supporting Information

Universal Reversible Hydrogen Potential for Electrocatalytic Ammonia Splitting Reactions in Non-Aqueous Solvents from Unified pH Measurements

Chenjia Mi[#,a], Jaan Saame[#,b], Agnes Heering,[b] Xiaoyin Zhang,[a] Oluwafemi Abubakar,[a] Ivo Leito,*[,b] and Thomas W. Hamann*[,a]

[#] Authors contributed equally to this work

[a] *Department of Chemistry, Michigan State University, East Lansing, Michigan 48824-1322 (USA)*

[b] *Institute of Chemistry, University of Tartu, Ravila 14a Str, 50411 Tartu (Estonia)*

*ivo.leito@ut.ee

*hamann@msu.edu

Experimental Methods

Open Circuit Potential Measurements

Solvent Purification and Storage

Acetonitrile (MeCN) was distilled from an alumina column dry still (to remove water), then distilled from concentrated sulfuric acid (99.999% metal bases, from Sigma Aldrich, b.p. = 81.6 – 82.2 °C. The as-distilled solvent was stored over activated 3 Å molecular sieves (see below for activation procedure). The water concentration was measured with Karl-Fischer titrator after every OCP titration experiment to be 5 – 15 ppm.

Tetrahydrofuran (THF) was distilled from sodium and was stored over activated 3 Å molecular sieves. The water concentration was measured with Karl-Fischer titrator after every OCP titration experiment to be 25 – 50 ppm.

N,N-Dimethylformamide (DMF) was distilled from calcium hydride under reduced pressure. The as-distilled solvent was stored over activated 3 Å molecular sieves.

Propylene carbonate (PC) was distilled with fractional distillation. The as-distilled solvent was stored over activated 3 Å molecular sieves. The water concentration was measured with Karl-Fischer titrator after every OCP titration experiment to be <5 ppm.

Chemical Synthesis and Purification

Molecular sieves (3 Å) were purchased from Sigma Aldrich, activated by heating to 160 °C under high vacuum for 12 h, stored in a sealed bottle in an oven (90 °C). Before use, the sieves are swirled with small amount of corresponding dry solvents for once (the solvents used here are then discarded) to remove dust.

Hydrogen (H_2, anhydrous) was purchased from Airgas and passed through a drying column packed with Drierite.

Ammonia (NH_3, anhydrous) was purchased from Airgas and passed through a drying column packed with calcium oxide before use.

Standard aqueous buffer solution (*p*H = 7) was purchased from VWR and used without further purification.

Trifluoromethanesulfonic acid (triflic acid, HOTf) wa purchased from Oakwood, stored in a freezer (-18 °C) and used without further purification.

Ammonium triflate (NH_4OTf) was synthesized by bubbling NH_3 gas through cold HOTf THF solution (~1M). Gaseous NH_3 was lead to the cold HOTf solution through a glass tube until excess, with the solution being vigorously stirred. The synthesis was of much larger scale (~1 kg NH_4OTf was produced). After synthesis, dry N_2 was bubbled through the reaction flask for 30 min to remove excess NH_3. The solvent THF was then removed *in vacuo* to yield a white (with some pale-yellow color) solid. The product was then split into 5 batches and each batch was recrystallized with THF, dried under high vacuum over 72 h, and stored in a sealed bottle under N_2 atmosphere. ^1H NMR (500 MHz, CD_3CN) δ 6.16 (s, 4H); ^{19}F NMR (500 MHz, CD_3CN) δ -79.54.

Tetrabutylammonium hexafluorophosphate (Bu_4NPF_6) was purchased from Alfa Aesar and recrystallized 3 times with 200 proof ethanol. The product was dried under high vacuum over 72 h and stored in a brown glass bottle. ^1H NMR (500 MHz, CDCl3) δ 3.21 – 3.05 (m, 2H), 1.67 – 1.50 (m, 2H), 1.41 (h, *J* = 7.4 Hz, 2H), 0.99 (t, *J* = 7.3 Hz, 3H).

Ferrocene was purchased from Sigma Aldrich, sublimed and stored in a glass vial in a desiccator. ^1H NMR (500 MHz $CDCl_3$) δ 4.16 (s, 10H).

Decamethylferrocene was purchased from Sigma Aldrich, either recrystallized from diethyl ether and dried under high vacuum over 12 h, or sublimed under vacuum, and the product was stored in a glass vial in a desiccator. ^1H NMR (500 MHz $CDCl_3$) δ 1.69 (s, 30H).

Silver nitrate and silver triflate are purchased from Oakwood and used without further purification.

Silver wire was purchased from Sigma Aldrich and polished with sandpaper before use.

Preparation of Electrolyte and Titrant

Electrolytes are prepared with 4 different solvents described above, with NH_4OTf as supporting electrolyte. To the electrolyte solutions a suitable internal reference was added. The components and concentrations of electrolytes are listed in Table S1.

The as-prepared electrolyte was used to fill the cell, to fill the secondary junction tube of the double junction RE, and to dissolve the acid/base to make titrant solutions.

Table S1. Electrolyte and Titrant Information in OCP Titration Experiments with NH_3-NH_4OTf Base-Acid Pair

Solvent	Supporting Electrolyte	Titrant[b]	Internal Reference
**MeCN**	NH_4OTf, 0.3 M[a]	NH_3-NH_4OTf 2:1 eurefstic mixed with solvent, equivalently 1 M NH_4OTf	$Me_{10}Fc$
**THF**	NH_4OTf, 1 M	NH_3-NH_4OTf 2:1 eurefstic mixed with solvent, equivalently 1 M NH_4OTf	$Me_{10}Fc$
**DMF**	NH_4OTf, 1 M	NH_3-NH_4OTf 2:1 eurefstic mixed with solvent, equivalently 1 M NH_4OTf	$Me_{10}Fc$
**PC**	NH_4OTf, 1 M	NH_3-NH_4OTf 2:1 eurefstic mixed with solvent, equivalently 1 M NH_4OTf	Fc[c]

[a] Using lower concentration due to solubility issue.

[b] Only base titration was performed, as the acid was also the supporting electrolyte. To obtain a solution at BE condition, NH_3 gas can be directly bubbled into the solution before measuring OCP.

[c] Did not use $Me_{10}Fc$ due to poor solubility.

Apparatus and Assembly

The OCP cell was a homemade cell (see Figure S1): a glass cylindrical cell with ~10 mL volume (maximum 15 mL), with a 14/20 glass joint on top (vertical) for adapting condenser, 3 threaded necks (cut from thermometer adaptors and fused to the cell) on the side (~60° angle) adapt to plastic screw caps for electrodes, 2 valved tubes on the side as gas outlets, and a specially-designed glass tube (~45 ° angle) that goes inside the cell with a small hole close to its tip and directly pointing towards an electrode (if inserted from one of the treaded necks) as H_2 inlet. The tip of the H_2 tube was spaced from the bottom of the cell so that a micro stir bar can move freely in the bottom of the cell.

Pt disk electrode was purchased from BASi. Each time before use, the Pt disk electrode was polished with alumina-water slurry on a polishing pad for 5 min, rinsed with water, sonicated in water for 30 s. Then the Pt disk electrode was set in a cell with 0.1 M K_2SO_4 solution, electrochemically conditioned by applying -2.5 V *vs.* a Pt mesh counter electrode to run 2-electrode chronoamperometry for 1800 s without stirring. Then the Pt disk was rinsed with flowing water for 2 min, sonicated in water for 30 s, and dried in N_2 flow.

Glassy carbon (GC) disk electrode was purchased from BASi and cleaned similarly to Pt disk electrode, except for the conditioning step. For thorough cleaning, the glassy carbon electrode can be set in a cell with 1 M H_2SO_4 solution and run 2-electrode cyclic voltammetry (CV) from -2.5 V to 1.5 V *vs.* a Pt mesh counter electrode at 250 mV/s scan rate for 100 cycles, followed by extensive cleaning with water and drying in N_2 flow.

The $Ag^{0/+}$ reference electrode (RE) was homemade: 0.01 M $AgNO_3$ and 0.1 M Bu_4NPF_6 was dissolved in MeCN to make a stock solution; to a glass tube (RE tube) with a magnesia frit on one narrow end, ~1 mL of the $AgNO_3$ stock solution was filled from the other open end; a Ag wire was polished and pierced through a rubber septum and soaked in the solution in the RE tube, with the septum sealing the open end of the RE tube. Loctite EA1C epoxy was used to help sealing the Ag wire with the rubber septum to reduce the probability of leaking. The RE was stored by soaking the frit end into the $AgNO_3$ stock solution in a flask and the flask was sealed with a thermometer adaptor and the RE tube to prevent evaporation of the solvent, and the flask was stored in a dark drawer. Before first use the RE was rested for 24 h to allow $Ag^{0/+}$ to reach equilibrium..

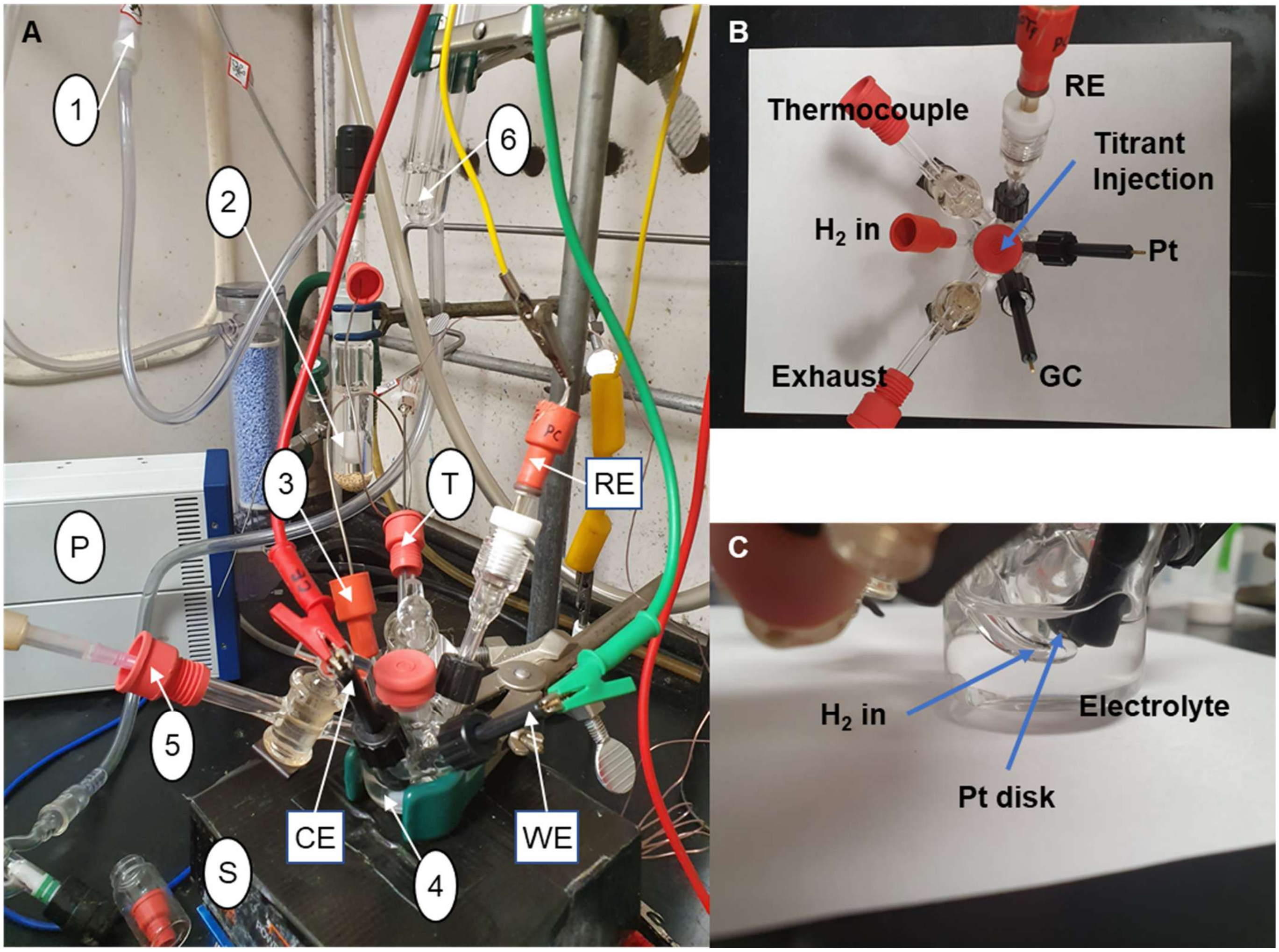

Figure S1. (A) Picture of the OCP cell setup for OCP measurements. Label meaning: WE: Pt disk working electrode; RE: reference electrode; CE: glassy carbon (GC) counter electrode; P: Gamry potentiostat; S: stirrer; T: thermocouple (pierced through the septum); 1: H_2 line drying column; 2: H_2 line gas washer; 3: H_2 inlet (a canula pierced through the septum and lead H_2 to a glass tube); 4: electrolyte with a stir bar; 5. exhaust gas outlet; 6: exhaust bubbler. **(B)** Top view of the cell when disconnected from the setup. **(C)** Zoom-in view of the glass tube H_2 inlet and its relative position to the Pt disk electrode.

The $Ag/Ag(NH_3)_2OTf$ RE was made similarly: ~0.01 M AgOTf and NH_3-NH_4OTf eurefstic (2:1 mole ratio) was dissolved in corresponding solvent to make a stock solution (with equivalent NH_4OTf concentration being 1 M). Other steps are the same as making $Ag^{0/+}$ RE except the frit are made by fusing Pt wire to the RE tube instead of magnesia. The $Ag/Ag(NH_3)_2OTf$ RE was used for OCP titration experiments with NH_3-NH_4OTf base-acid pair.

When used for OCP experiments, the RE was assembled with a secondary junction to make a double-junction RE: the primary junction was the as-assembled $Ag^{0/+}$ or $Ag/Ag(NH_3)_2OTf$ RE, and the secondary junction was a bigger glass tube with a magnesia frit on one end and threaded opening on the other end; to this tube ~1 mL of the corresponding electrolyte was filled; the primary RE tube was inserted into this tube and sealed with an O-ring and a Teflon screw cap on the threaded end. The frit of the primary RE was soaked into the electrolyte solution inside the secondary junction tube, while the frit of the secondary junction tube was soaked into the bulk electrolyte when assembled to the cell.

To assemble the cell, each electrode – Pt disk working electrode (WE), double junction RE and glassy carbon counter electrode (CE) – was mounted with an O-ring and set through a threaded neck on the cell. Specifically, the Pt disk WE should be the one directly pointed by the H_2 gas inlet tube. 10.00 mL of electrolyte solution was added to the cell with a micro stir bar. The vertical 14/20 joint was typically sealed with a rubber septum to facilitate OCP titration; if necessary, it can be mounted to a dry ice condenser (and in this case, the condenser was the gas outlet). H_2 inlet tube and both gas outlet tubes are sealed with a rubber septum. A needle-like thermocouple was pierced through the septum and the valve of one gas outlet and reaching the bulk electrolyte. A needle was pierced through the septum on the other gas outlet to lead the exhaust to a silicone oil bubbler. Dry H_2 gas was passed through a bubbler-sized gas washer containing corresponding dry solvent and lead into the H_2 inlet with a cannula with both ends piercing through rubber septa – so that the entire system was closed from the atmosphere except the exhaust bubbler. If a condenser was used, close the valve on the unused gas outlet tube. If NH_3 flow was needed (see below), it can be lead in through one of the gas outlets. If a low (or high) temperature was desired, a bath can be set outside of the cell. After assembled the cell was clamped above a magnetic stirrer and adjusted so that the stir bar can spin steadily inside the cell.

OCP Calibration and Measurements

To calibrate the effective H_2 partial pressure p_{H2}, a pH = 7 standard aqueous buffer solution was used as electrolyte, and a Ag/AgCl RE with saturated KCl solution (double-junction with the pH = 7 buffer solution) was used. H_2 passed through the gas washer containing water was lead to the cell and bubbled over the Pt disk electrode. OCP between the Pt disk WE and the RE was monitored (see below) until stable (no systematical inclining or declining trend and variation was within 1 mV over 120 s) and this stable OCP was recorded as $E_{\text{OCP } vs. \text{ Ag/AgCl}}$. Note that each time when the H_2 flow rate was changed the calibration was redone. The Ag/AgCl RE was then calibrated against a Gamry standard Ag/AgCl RE, and the calculation formula was as follows:

$$E_{corr} = \frac{2.303RT}{F} log\, p_{H_2}^{1/2} = -(E_{OCP\_vs\_\text{Ag/AgCl}} - E_{\text{NHE}\_vs\_\text{Ag/AgCl}}) - \frac{2.303RT}{F} pH$$

where E_{corr} was the H_2 activity correction term in V and was independent of references; $E_{OCP\_vs\_\mathrm{Ag/AgCl}}$ was the experimental OCP measured against the Ag/AgCl RE; $E_{\mathrm{NHE}\_vs\_\mathrm{Ag/AgCl}}$ was the formal potential of proton reduction in water (NHE) against the Ag/AgCl RE.

To measure OCP in general, an electrolyte solution was prepared with solvent of interest and supporting electrolyte if needed. Dry H_2 passed through the gas washer containing corresponding dry solvent was lead to the cell and bubble over the Pt disk electrode, with its flow rate unchanged from the previous calibration step (here we assume the viscosity difference between water and solvent of interest has negligible impact on the effective p_{H2}). The Pt WE and the RE are connected to the potentiostat, while the CE will not be used in OCP measurements (even if connected). Stirring of the electrolyte was turned on. OCP was measured until stable. This stable value (averaged over a set period of time) was recorded as $E_{OCP\_vs\_\mathrm{RE}}$.

To perform an OCP titration experiment, the cell was setup and OCP between WE and RE reached a stable value with only the electrolyte. The titrant was then transferred to a graduated syringe with a stop valve. The syringe was pierced through the rubber septum on top of the cell into the electrolyte solution. With continuous OCP measurement (while maintaining the H_2 bubbling and stirring), aliquots of titrant was injected into the electrolyte, with the volume accurately read from the syringe. After each injection, the cell was allowed to reach equilibrium, monitored by a stabilizing OCP. Once a stable OCP was reached, a sampling time (usually 30 s for non-volatile acid/base and 10 s for volatile acid/base) was given. The temperature of the solution was recorded through the thermocouple. After sampling, the next aliquot was titrated into the cell and this procedure was repeated until all titrant was injected. A staircase diagram will show on the as-measured OCP data, with each "stair" yields a datum. The $E_{OCP\_vs\_\mathrm{RE}}$ was determined by averaging the collected data within each sampling time ("stair"), and its uncertainty was given by the standard deviation within each sampling time. These $E_{OCP\_vs\_\mathrm{RE}}$ values depict a function of ln([B]/[A]) as described by Nernst equation.

Before, in between and after OCP titration experiments, CV of internal reference (Fc or $\mathrm{Me_{10}Fc}$) was measured to determine the RE potential. To measure CVs, the glassy carbon electrode was connected to the WE of the potentiostat, while the Pt disk electrode was used as CE, and RE connection was the same as in OCP experiments. CV was usually measured at 0.1 V/s scan rate for 3 cycles. It was typically observed that using the Pt disk as CE does not foul its surface, while using it as WE should be avoided if further OCP measurements will be performed in the same cell. The CV of Fc or $\mathrm{Me_{10}Fc}$ has not been seen to change with any addition of acid/base, including ammonia, to the highest concentration listed in Table 1 and 2. The redox potential of the internal reference, $E_{1/2,\mathrm{InRef}}$, was obtained from CV, and the as-measured $E_{OCP\_vs\_\mathrm{RE}}$ shall be converted to $E_{OCP\_vs\_\mathrm{InRef}}$ (where "InRef" subscript stands for internal reference) for comparison purpose using the following equation:

$$E_{OCP\_vs\_\mathrm{InRef}} = E_{OCP\_vs\_\mathrm{RE}} - E_{1/2,\mathrm{InRef}}$$

Note that in this process, any liquid junction potential (LJP) between the RE and the electrolyte was cancelled as the LJP impacts OCP and CV measurements equally in the same cell. Since $\mathrm{Me_{10}Fc}$ was used as internal reference in most cases, $E_{OCP\_vs\_\mathrm{Me10Fc}}$ will be written as E_{OCP} for simplicity, while $E_{OCP\_vs\_\mathrm{Fc}}$ will be specified as-is.

The actual concentration of the acid, [A], and the base, [B], in the cell was calculated from the titrant stock solution concentration and titrant volume (see Table S3 and S4), accounting for the electrolyte volume change caused by adding titrant (assuming the volume of electrolyte and titrant are additive). In the case of

NH_3-NH_4OTf titration experiment, 50 μL of the titrant stock solution was added to 50 mL H_2O, and then titrated with 50 mM aqueous HCl with 3 drops of methyl orange indicator to determine the NH_3 concentration. The titration point was reached once the solution turns from yellow to red.

If OCP at NH_3 bulk electrolysis (BE) condition needs to be measured (usually after OCP titration), NH_3 gas was directly bubbled through the cell solution for 5 min with stirring, while the temperature of the solution and the OCP between the Pt|H_2 WE and the RE was monitored. The temperature usually rises due to the association between NH_3 and NH_4OTf when starting bubbling NH_3, and then slowly drops after NH_3 was saturated. Once saturation was reached (judged from the temperature and the OCP reading), the NH_3 gas inlet was pulled out from the solution to stop NH_3 bubbling but still allow NH_3 to flow through the head space, in order to maintain an NH_3 saturation condition (note that when the temperature decreases, more NH_3 can be dissolved). The $E_{OCP\_vs\_\mathrm{RE}}$ was recorded after the temperature falls back to room temperature for a sampling time of 120 s. Note that with continuous H_2 bubbling the temperature of the solution may drop below room temperature, and a bath can be used to maintain a stable temperature if desired. After OCP measurements, CV of internal reference was measured, and 100 μL of the electrolyte solution was taken to perform an acid-base titration with aqueous HCl described above to determine the final NH_3 concentration.

Table S2. A Typical Data Sheet for OCP Titration with NH_3-NH_4OTf

Titrant Volume (μL)	Total Volume (μL)	[NH_3] (M)	[NH_4OTf] (M)
0	10000	0.0	1
10	10010	0.002	1
30	10030	0.006	1
50	10050	0.010	1
100	10100	0.020	1
150	10150	0.030	1
200	10200	0.039	1
250	10250	0.049	1

The total volume was assumed to be unchanged upon dissolving extra NH_3.

The NH_3 concentration of the final solution was determined by titration with HCl.

Calibration of OCP Cell

Figure S2 shows an example of OCP calibration (note that every time when the H_2 line or the OCP cell configuration was changed, a new calibration was needed). As shown in Figure 2A, stirring the electrolyte does not change the average OCP value but only adds a sine-wave noise, compared to that measured without stirring. To ensure fast and thorough mixing of titrants and electrolytes, in the later OCP measurements stirring was kept on. From this experiment E_{corr} = -22.6 ± 0.2 mV was obtained at 295.45 K.

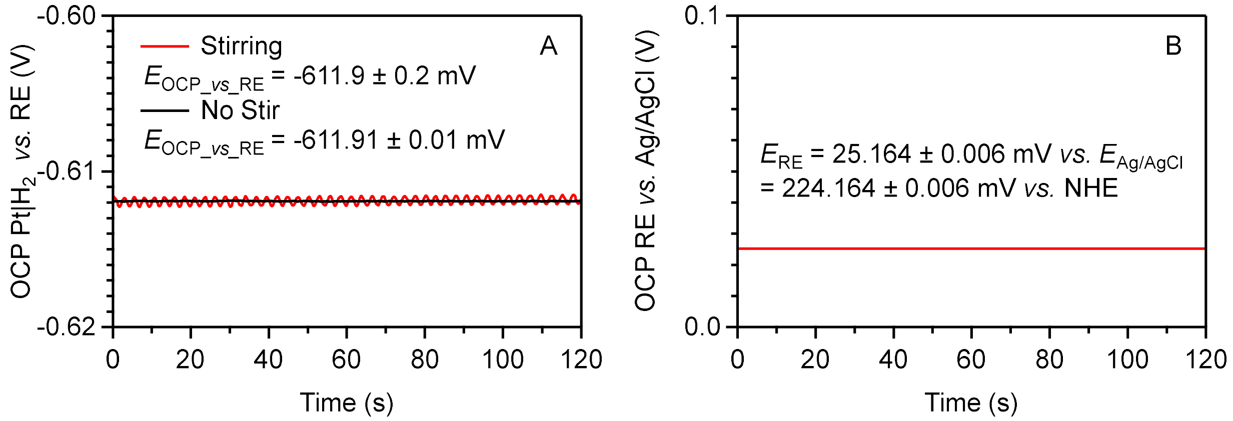

Figure S2. (A) Calibration of OCP in a pH = 7.00 buffer solution between the Pt|H_2 WE and a home-made Ag/AgCl double junction RE. The OCP was recorded with (red) and without (black) stirring the electrolyte. The $E_{OCP\_vs\_RE}$ value in each condition was shown. **(B)** OCP between the home made RE and a commercial Ag/AgCl electrode (from Gamry, with a nominated potential of 199 mV *vs.* NHE).

OCP Results

Figures S3-S5 show the results of OCP titrations with NH_3-NH_4OTf base-acid pair in THF, DMF and PC. Results are discussed in the manuscript.

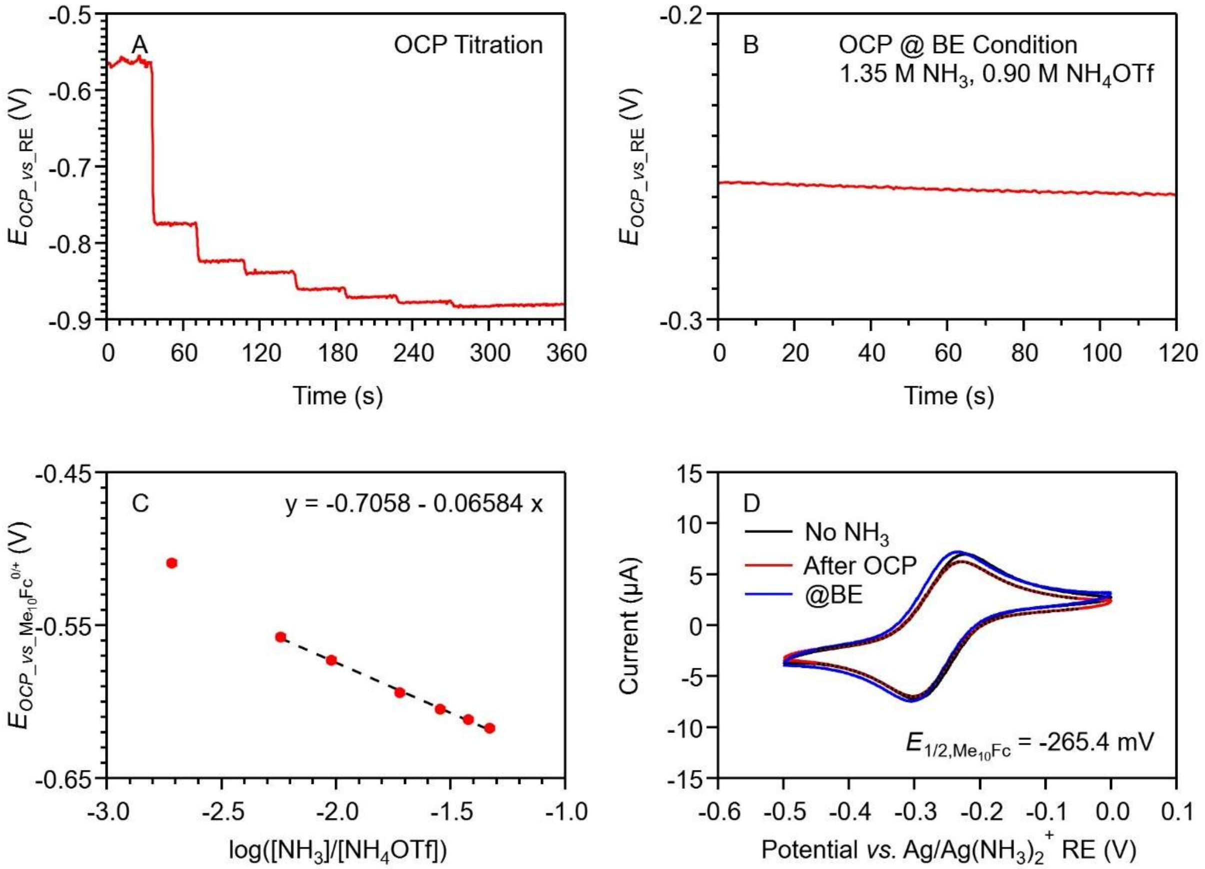

Figure S3. (A) OCP titration of NH_3-NH_4OTf base-acid pair in THF. Each "stair" was recorded as an OCP datum with a corresponding concentration of NH_3. **(B)** OCP at BE condition. **(C)** Nernst analysis of OCP titration data. **(D)** CV of internal reference $Me_{10}Fc$ in the same cell.

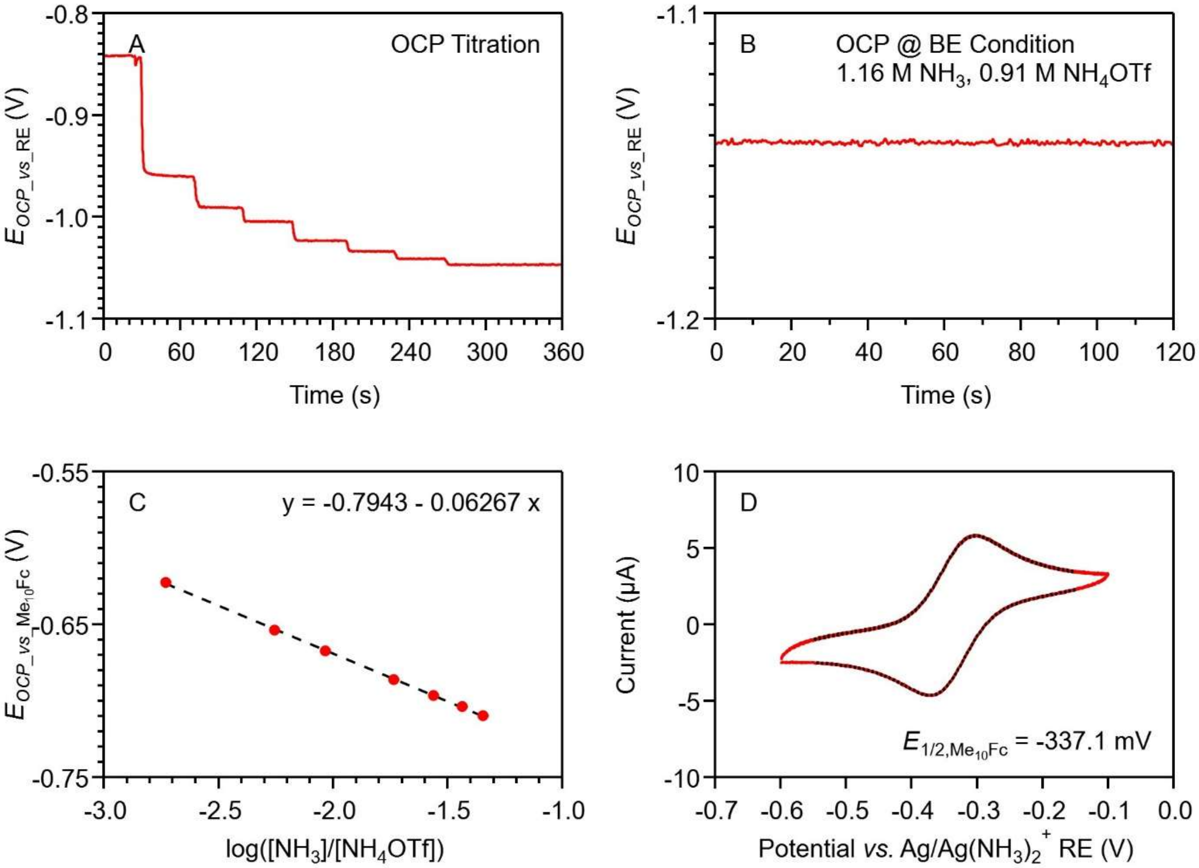

Figure S4. (A) OCP titration of NH_3-NH_4OTf base-acid pair in DMF. Each "stair" was recorded as an OCP datum with a corresponding concentration of NH_3. **(B)** OCP at BE condition. **(C)** Nernst analysis of OCP titration data. **(D)** CV of internal reference $Me_{10}Fc$ in the same cell.

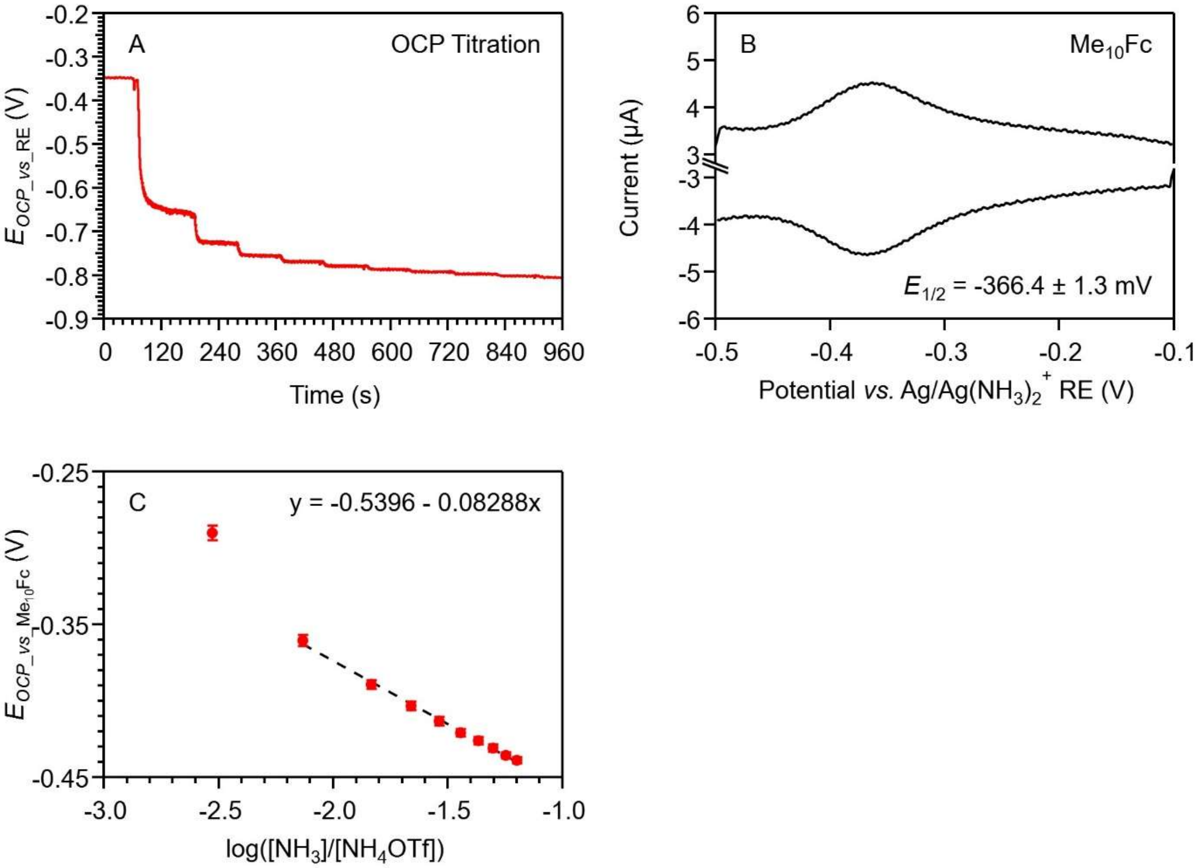

Figure S5. (A) OCP titration of NH_3-NH_4OTf base-acid pair in PC. Each "stair" was recorded as an OCP datum with a corresponding concentration of NH_3. **(B)** SWV of internal reference $Me_{10}Fc$ in the same cell, scanned on both directions. **(C)** Nernst analysis of OCP titration data.

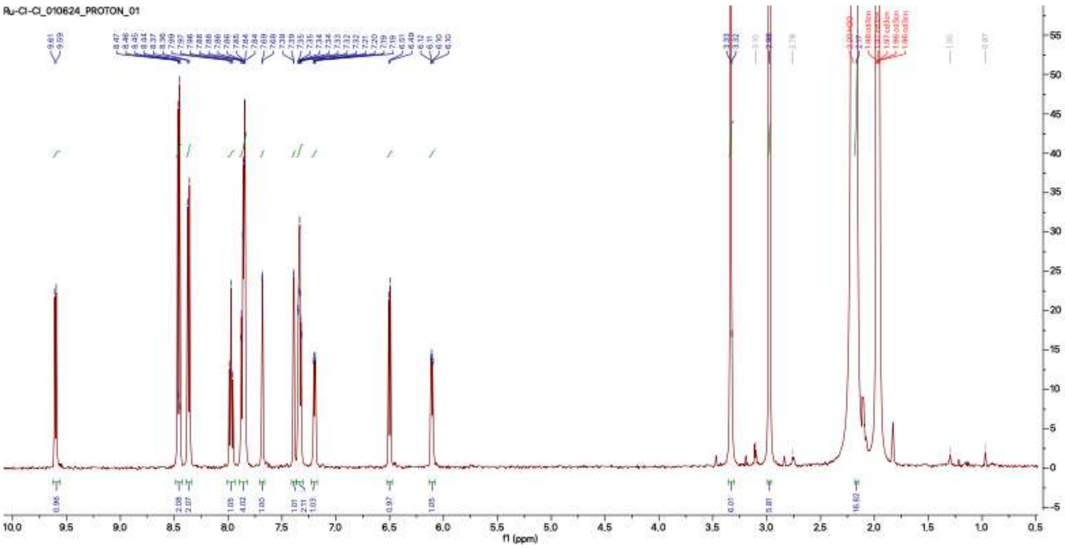

Figure S6. ^{1}H NMR spectra of [Ru(tpy)(dmabpy)Cl]Cl in MeCN-d_3

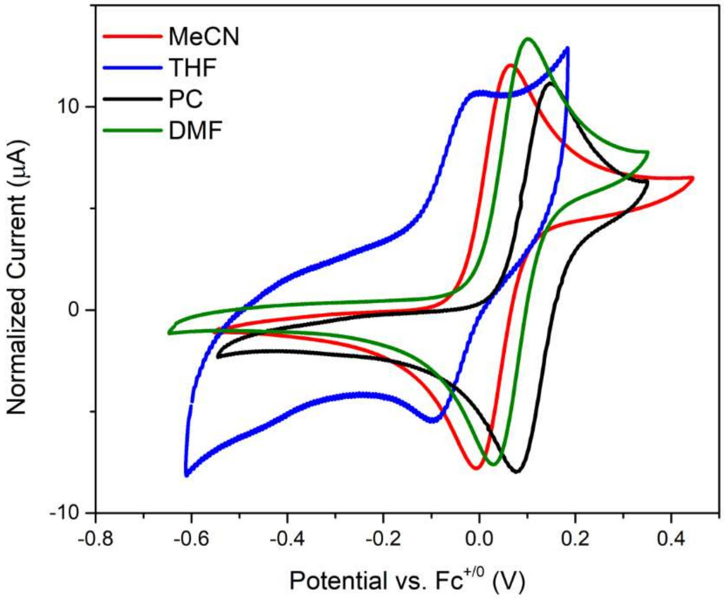

Figure S7. Cyclic voltammograms of 2.5 mM [Ru(tpy)(dmabpy)Cl]$^{+}$ measured at a scan rate of 10 mV/s in MeCN (red), THF (blue), PC (black), and DMF (green) solutions containing 0.1 M NH_4OTf as a supporting electrolyte.

Table S3. Uncompensated resistance of 0.1 M NH_4OTf solution in different solvents. Values were derived from the intercept of fitted electrochemical impedance measurements. The relatively large resistances are attributed to the high impedance CE utilized which exhibits stable potential in all solvents and is ultra no-leak.

Solvent	Resistance (Ω)
MeCN	204.8
THF	3879
PC	869.3
DMF	208.6

Table S4 Conductivity of 0.1 M NH_4OTf solutions as a function of solvent. The additional trend in uncompensated resistance, in addition to the high impedance reference, can be attributed to differences in electrolyte conductivity.

Solvent	Conductivity (μS/cm)
MeCN	5550
THF	133.0
PC	2136
DMF	6480

Table S5. Measured $E_{½}$ values of $Fc^{+/0}$ vs homemade AgCl/Ag reference electrode

Solvent	$E_{1/2}$ *vs* AgCl/Ag (V)
MeCN	0.288
THF	0.414
PC	0.361
DMF	0.379

Table S6. Anodic and cathodic peak separation (ΔE) and $E_{½}$ values of $[Ru(tpy)(dmabpy)Cl]^{+}$ for the solvents listed in column 1, taken from CVs in Figure S7.

**Solvent**	ΔE **(V)**	$E_{1/2}$ ***vs*** $\mathbf{Fc^{+/0}}$ **(V)**	$\mathbf{E_{cat/2}}$ **vs** $\mathbf{Fc^{+/0}}$ **(V)**
MeCN	0.073	0.100 ± 0.005	0.105
THF	0.089	-0.059 ± 0.007	
PC	0.078	0.117 ± 0.007	0.096
DMF	0.075	0.077 ± 0.02	0.078

Measurements of NH_3 solubility

The procedure described in prior literature was followed.[1]

10 mg of 4-cyanoanilinium tetrafluoroborate was dissolved in 0.6 ml of MeCN-d3 and transferred to a GC vial. 1.5 ml of the dry solvent was transferred into a separate GC vial and sparged with ammonia for 30 minutes. 10 μL of the saturated ammonia solution was transferred into the prior GC vial and the solution was shaken gently. 7 μL of hexamethyldisiloxane (HMDSO) was added to the vial as a standard with 18 equivalent protons, and the mixture was transferred to an NMR tube. The ^{1}H NMR peaks of NH_4^+, a 1:1:1 triplet at 5.96 ppm, were integrated and referenced to HMDSO. The concentration of ammonia was calculated using the formula:

$$[\mathrm{NH_3}] = \frac{integration\ of\ \mathrm{NH_4^+}}{integration\ of\ \mathrm{HMDSO}} \times \frac{18}{4} \times \frac{3.3\times\ 10^{-5}\ mol}{1\times 10^{-5}\ L}$$

Table S7. Measured concentrations of saturated NH_3 in the non-aqueous solvents listed in column 1. The uncertainty is taken as the standard deviation of three independent measurements.

**Solvent**	**Trial 1 (M)**	**Trial 2 (M)**	**Trial 3 (M)**	**Average Conc (M)**
MeCN	1.56	1.66	1.77	1.7 ± 0.1
PC	1.96	1.97	1.96	1.96 ± 0.01
THF	2.39	2.47	2.42	2.43 ± 0.04
DMF	2.93	3.42	3.48	3.3 ± 0.3

The solvation energy can then be calculated by: $\Delta G = -RT\ \ln(K_{eq})$

Where R is 0.001987 kcal·mol^{-1}K^{-1}, T is 298 K and $K_{eq} = \frac{[\mathrm{NH_3}]}{1\ atm}$

Table S8. Calculated solvation energy of NH_3 in the non-aqueous solvents listed in column 1, with the associated potential correction due to the solvation: $NH_3(g) \rightarrow NH_3(solv)$. The uncertainty estimate is derived from the uncertainty of the saturated concentration measurements.

**Solvent**	SolvationEnergy (kcal/mol)	Solvation Potential Correction (mV)
MeCN	-0.30 ± 0.03	4
PC	-0.40 ± 0.02	6
THF	-0.52 ± 0.01	8
DMF	-0.71 ± 0.05	10

References

(1) Lindley, B. M.; Appel, A. M.; Krogh-Jespersen, K.; Mayer, J. M.; Miller, A. J. M. Evaluating the Thermodynamics of Electrocatalytic N_2 Reduction in Acetonitrile. *ACS Energy Letters* **2016**, *1* (4), 698-704. DOI: 10.1021/acsenergylett.6b00319.
